# Prognostic Impact of Perioperative CA125 Status in Gastric Cancer Based on New Cutoff Values

**DOI:** 10.7759/cureus.61609

**Published:** 2024-06-03

**Authors:** Jin Moriyama, Hideaki Shimada, Yoko Oshima, Takashi Suzuki, Satoshi Yajima, Fumiaki Shiratori, Kimihiko Funahashi

**Affiliations:** 1 Department of Surgery, Moriyama Hospital, Kanagawa, JPN; 2 Department of Surgery and Clinical Oncology, Toho University, Tokyo, JPN; 3 Department of Surgery, Toho University, Tokyo, JPN

**Keywords:** carbohydrate antigen 125 (ca125), surgery, prognosis, recurrence, gastric cancer

## Abstract

Objectives

The current carbohydrate antigen 125 (CA125) cutoff value demonstrated high specificity but low sensitivity. Therefore, we used new cutoff values to evaluate the clinical impact of perioperative CA125 in gastric cancer.

Methods

This study retrospectively analyzed 525 patients with gastric cancer (349 males and 176 females), of whom 445 patients underwent R0 resection and 80 patients underwent R1/R2 resection between 2011 and 2020. The receiver operating characteristic curve indicated preoperative and postoperative cutoff CA125 values of 15.7 IU/mL and 17.3 IU/mL, respectively, to predict overall survival. Furthermore, we analyzed changes in postoperative CA125 levels and evaluated their prognostic impact using multivariate analysis.

Results

The preoperative CA125-positive rate was 25%. Males, advanced TNM factors, and noncurative resection cases demonstrated significantly higher positive rates than the other group. The preoperative CA125-positive group exhibited a significantly higher noncurative resection rate than the preoperative CA125-negative group (32% versus 10%, P < 0.01). Preoperatively, CA125-positive status was an independent poor prognostic factor (P < 0.01), and at three months postoperatively, it tended to be a poor prognostic factor.

Conclusions

High preoperative CA125 (>15.7 IU/mL) was a significant predictor for noncurative resection and poor overall prognosis in gastric cancer. Furthermore, postoperative CA125-positive status three months postoperatively was also a potential predictor of recurrence and poor prognosis.

## Introduction

Treatment outcomes and long-term prognosis for advanced and recurrent gastric cancer have improved with the advancement of new drug therapies [1,2]. However, the treatment response for peritoneal dissemination cases remains extremely poor [3,4]. Various factors are known to be associated with poor prognosis, including high levels of tumor markers such as carcinoembryonic antigen (CEA) and carbohydrate antigen 19-9 (CA19-9), tumor progression status, depth of invasion, and lymph node metastasis [5]. Most reports have revealed carbohydrate antigen 125 (CA125) as a poor prognostic factor associated with peritoneal dissemination [6-10]. Previous studies on the clinicopathological significance of CA125 in patients with gastric cancer have included nonsurgical cases [8,9]. Only a few reports focused on the prognostic impact of perioperative CA125 changes [11,12].

Previous studies used a cutoff value that was determined based on the values of healthy subjects (>35 IU/mL) [8,11]. Because these reports mainly focused on patients with highly advanced gastric cancer, the reported sensitivities demonstrated a wide range (9%-34%). Conversely, the CA125 positive rate in early gastric cancer was extremely low at 1.9% [13]. In clinical practice, the positive rate of CA125 is inadequate to diagnose and predict the prognosis of patients with gastric cancer at various stages.

Therefore, this study proposed a new cutoff value and analyzed the clinicopathological and prognostic significance of CA125, the perioperative changes in CA125 values, and the prognostic impact of CA125 status at three months postoperatively.

## Materials and methods

**Figure 1:**
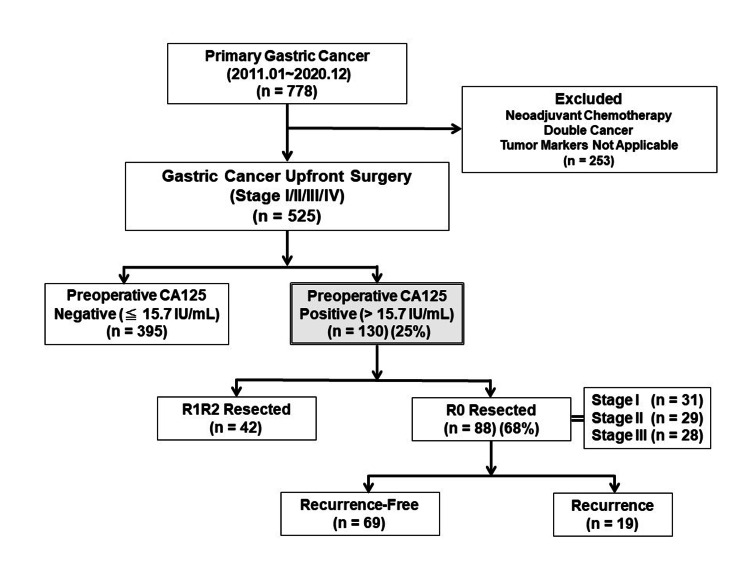
Flowchart of patient selection CA125, carbohydrate antigen 125; R0, no residual tumor; R1, microscopic residual tumor; R2, macroscopic residual tumor

Figure 1 shows a flowchart of patient selection. These medical records were obtained from the medical record database of Toho University Omori Hospital between January 2011 and December 2020. A total of 525 patients (349 males and 176 females, with a mean age of 69 years) were enrolled from 778 patients with primary gastric cancer underwent surgery. Patients who received neoadjuvant therapy, patients with double cancers, and patients with no tumor markers data were excluded. Standard surgical treatment was total or distal gastrectomy combined with D2 lymph node dissection according to the guidelines of the Japanese Gastric Cancer Society [14]. In addition, we performed bypass surgery, exploratory laparotomy, and exploratory laparoscopy for some stage IV patients as palliative surgery. Therefore, 445 patients underwent R0 resection and 80 patients underwent R1/R2 resection. The postoperative stage was classified into stages I to IV according to the clinicopathological classification based on the Japanese Gastric Cancer Classification (Third English Edition) [15]. As postoperative follow-up, tumor markers and imaging examinations were performed every three months, and the CA125 values were evaluated before the start of all treatments and three months after surgery. We divided them into positive and negative groups based on their respective cutoff values.

**Figure 2:**
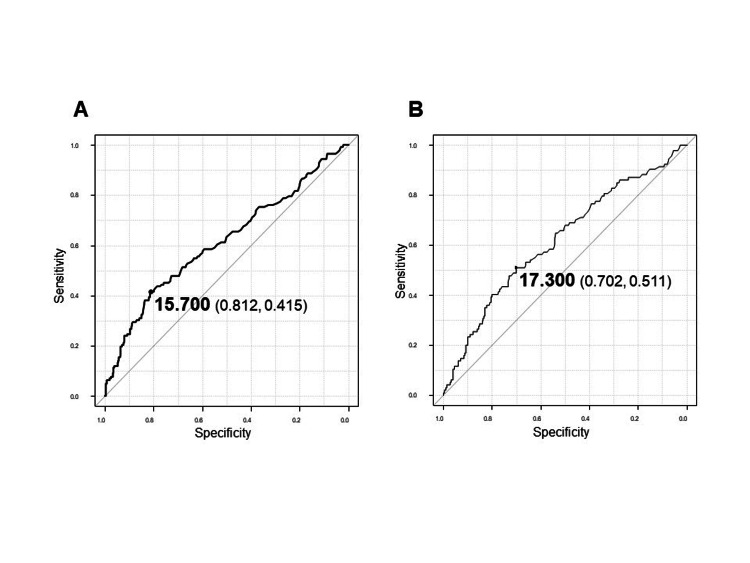
The receiver operating characteristic curves of (A) preoperative CA125 and (B) postoperative CA125 CA125, carbohydrate antigen 125

The preoperative and three months postoperative cutoff values of CA125 were 15.7 IU/mL and 17.3 IU/mL, respectively, to predict overall survival based on the receiver operating characteristic curve (Figure 2). The prognostic impact of preoperative CA125 was evaluated using multivariate analysis. Moreover, we analyzed the relationship among the change in CA125 status postoperatively, CA125 status at three months postoperatively, and prognosis. The ethics committee of Toho University Omori Medical Center approved this retrospective study (no. M20196 19056 18002).

Differences between groups were analyzed using Fisher’s exact probability test for the categorical variables. The Kaplan-Meier method was used to generate the overall survival curves, and the log-rank test was used to assess the differences. Overall survival was evaluated using univariate analysis with the log-rank test. A p-value of <0.05 indicated statistical significance. EZR (Saitama Medical Center, Jichi Medical University, Saitama, Japan), a graphical user interface of R (R Foundation for Statistical Computing, Vienna, Austria), was used for all statistical analyses. This software is an improved version of R Commander, designed to more accurately add statistical functions that are frequently used in biostatistics [16].

## Results

Background characteristics of patients

The preoperative CA125-positive (>15.7 IU/mL) group consisted of 130 (25%) patients (Figure 1), including 88 (68%) and 42 (32%) patients who underwent R0 and R1/R2 resection, respectively. Stage classification of the R0 resected group showed stages I, II, and III in 31 (35%), 29 (33%), and 28 (32%) patients, respectively. The recurrence group comprised 19 (21%) patients.

Comparison of clinicopathological variables with preoperative CA125 status in patients with gastric cancer

The preoperative CA125-negative (≤15.7 IU/mL) and CA125-positive groups comprised 395 (75%) and 130 (25%), patients, respectively (Table 1). Generally, advanced tumors (T4, N+, M+, P+, CY+, and H+ groups) showed CA125-positive status more frequently than the other group (P < 0.01). The preoperative CA125-negative group demonstrated a higher R0 resection rate than the CA125-positive group (90% vs. 68%) (P < 0.01). The R1/R2 resection rate was significantly higher in the preoperative CA125-positive group than in the CA125-negative group (32% vs. 10%) (P < 0.01).

**Table 1:** Comparison of clinicopathological variables with preoperative CA125 status in patients with gastric cancer *Fischer’s exact probability test CA125, carbohydrate antigen 125; T, depth of tumor invasion; R0, no residual tumor; R1, microscopic residual tumor; R2, macroscopic residual tumor

Factor	Group	CA125 negative		CA125 positive		P-value*
		395	100%	130	100%	
Age	<65 (134)	110	28%	24	18%	0.04
	≥65 (391)	285	72%	106	82%	
Gender	Female (176)	136	34%	40	31%	0.45
	Male (349)	259	66%	90	69%	
Depth of tumor invasion	T1 (223)	197	50%	26	20%	<0.01
	T2 (57)	45	11%	12	9%	
	T3 (106)	79	20%	27	21%	
	T4 (139)	74	19%	65	50%	
Lymph node metastasis	Negative (307)	259	66%	48	37%	<0.01
	Positive (218)	136	34%	82	63%	
Distant metastasis	Negative (453)	364	92%	89	68%	<0.01
	Positive (72)	31	8%	41	32%	
Peritoneal metastasis	Negative (483)	377	95%	106	82%	<0.01
	Positive (42)	18	5%	24	18%	
Peritoneal lavage cytology	Negative (428)	341	86%	87	67%	<0.01
	Positive (61)	26	7%	35	27%	
	Unknown (36)	28	7%	8	6%	
Hepatic metastasis	Negative (517)	394	99%	123	95%	<0.01
	Positive (8)	1	1%	7	5%	
Pathological stage	1 (253)	222	56%	31	24%	<0.01
	2 (103)	74	19%	29	22%	
	3 (97)	68	17%	29	22%	
	4 (72)	31	8%	41	32%	
Histological type	Differentiated (278)	216	55%	62	48%	0.34
	Poorly (240)	174	44%	66	51%	
	Others (7)	5	1%	2	1%	
Resection grade	R0 (445)	357	90%	88	68%	<0.01
	R1/R2 (80)	38	10%	42	32%	

Comparison of overall survival between the preoperative CA125-positive and CA125-negative groups

**Figure 3:**
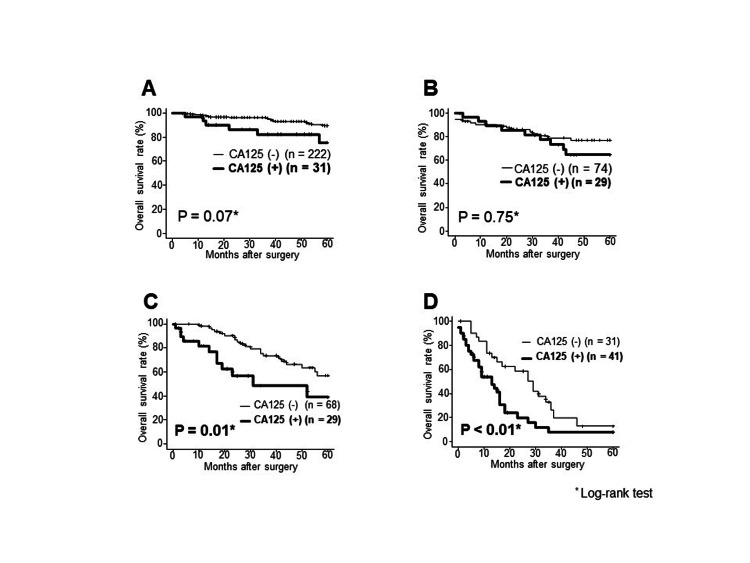
Comparison of overall survival between the preoperative CA125-positive and CA125-negative groups A. Stage Ⅰ. B. Stage Ⅱ. C. Stage Ⅲ. D. Stage Ⅳ. CA125, carbohydrate antigen 125

**Figure 4:**
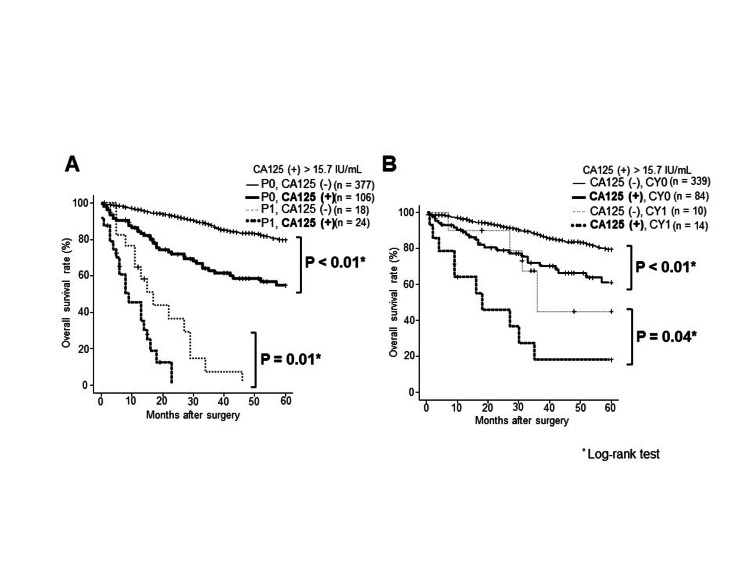
Comparison of overall survival between the preoperative CA125-positive and CA125-negative groups with peritoneal metastasis A. Preoperative CA125-positive and CA125-negative with P0 or P1. B. Preoperative CA125-positive and CA125-negative with CY0 or CY1. CY unknown: 36 cases excluded CA125, carbohydrate antigen 125; P0, no peritoneal metastasis; P1, peritoneal metastasis; CY0, peritoneal lavage cytology negative for carcinoma cells; CY1, peritoneal lavage cytology positive for carcinoma cells

The prognosis of the preoperative CA125-positive group with pathological stage III/IV was significantly worse than that of the CA125-negative group (P = 0.01) (Figure 3). The preoperative CA125-positive group demonstrated a significantly worse prognosis than the CA125-negative group despite peritoneal metastases (Figure 4A). Furthermore, the preoperative CA125-positive group without peritoneal metastasis exhibited a significantly worse prognosis than the CA125-negative group, although it was positive for ascites cytology (Figure 4B).

Table 2 shows the univariate and multivariate analyses of prognostic variables for overall survival in patients with gastric cancer. Male sex, pT3/T4, P1, H1, distant metastases, and preoperative CA125-positive status were poor prognostic factors in univariate and multivariate analyses (P < 0.01).

**Table 2:** Univariate and multivariate analyses of prognostic variables for overall survival in patients with gastric cancer *Log-rank test; ** Cox proportional hazard model CA125, carbohydrate antigen 125; M, male; F, female; pT, pathological depth of tumor invasion; P0, no peritoneal metastasis; P1, peritoneal metastasis; H0, no hepatic metastasis; H1, hepatic metastasis

	Univariate analyses*	Multivariate analyses**	
	P-value	Odds ratio (95% confidence interval)	P-value
Age (≥65/<65)	0.12	-	-
Gender (M/F)	0.02	1.67 (1.14, 2.45)	<0.01
Depth of tumor invasion (pT3T4/pT1T2)	<0.01	2.28 (1.50, 3.48)	<0.01
Peritoneal metastasis (P1/P0)	<0.01	8.31 (5.15, 13.42)	<0.01
Hepatic metastasis (H1/H0)	<0.01	4.50 (1.83, 11.01)	<0.01
Others distant metastasis (+)/(-)	<0.01	3.02 (1.68, 5.42)	<0.01
Histological type	0.27	-	-
(undifferentiated/differentiated)			
Preoperative CA125 status (+)/(-)	<0.01	1.81 (1.26, 2.60)	<0.01

Comparison of perioperative changes in CA125 levels until 18 months postoperatively between the recurrence and recurrence-free groups

**Figure 5:**
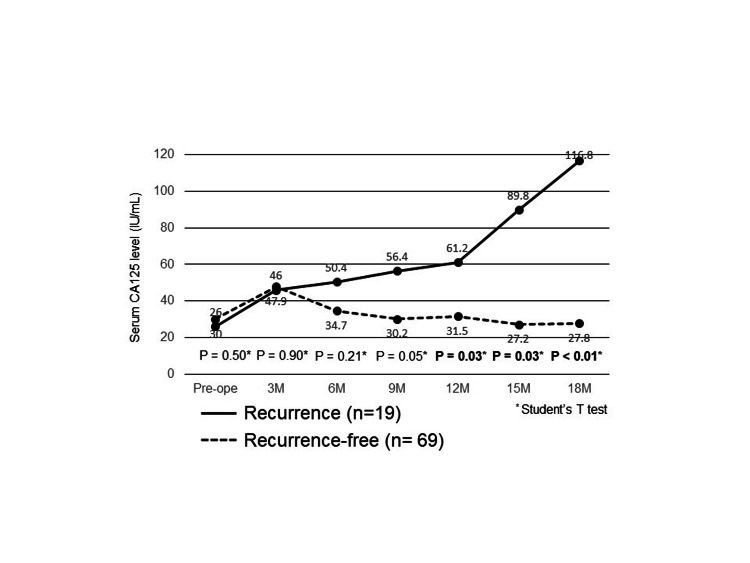
Comparison of perioperative changes in CA125 levels until 18 months postoperatively between the recurrence and the recurrence-free groups CA125, carbohydrate antigen 125; Pre-ope, preoperative; M, months

Figure 5 shows the perioperative changes in CA125 levels until 18 months postoperatively in the recurrence and recurrence-free groups. The two curves began to separate at three months postoperatively. These two curves were significantly different at 12 months postoperatively (P = 0.03).

Univariate and multivariate analyses of the prognostic impact of CA125 status at three months postoperatively on overall survival

The three months postoperative CA125-positive group showed significantly worse overall survival than the three months postoperative CA125-negative group (P = 0.03, Table 3). However, three months postoperative CA125 status was not an independent poor prognostic factor in the multivariate analyses (P = 0.08).

**Table 3:** Univariate and multivariate analyses of the prognostic impact of CA125 status at three months postoperatively on overall survival *Log-rank test; **Cox proportional hazard model; ***CA125(+) > 17.3 IU/mL CA125, carbohydrate antigen 125; M, male; F, female; pT, pathological depth of tumor invasion; pN, pathological lymph node metastasis

	Univariate analyses*	Multivariate analyses**	
	P-value	Odds ratio (95% confidence interval)	P-value
Age (≥65/<65)	0.22	-	-
Gender (M/F)	0.05	1.54 (0.79, 2.99)	0.19
Depth of tumor invasion	<0.01	1.41 (0.74, 2.67)	0.3
(pT3T4/pT1T2)			
Lymph node metastasis	<0.01	2.17 (1.14, 4.15)	0
(pN+/pN-)			
Histological type	0.36	-	-
(undifferentiated/differentiated)			
Three months postoperative CA125 status (+)/(-)***	0.03	1.63 (0.93, 2.84)	0.08

## Discussion

This study proposed a new cutoff value for CA125 and analyzed its clinicopathological and prognostic significance in gastric cancer patients who underwent surgical treatment, including conservative surgery. Preoperative CA125-positive status was an independent poor prognostic factor for overall survival. Moreover, we assessed the perioperative changes in CA125 values and the prognostic impact of CA125 status three months postoperatively. The difference was not statistically significant, although a CA125-positive status three months postoperatively appeared to be a poor prognostic factor.

Previous studies [8,10,11,17] used the cutoff values of CA125, which were determined based on the distribution of the values of the healthy group (Table 4). Those studies focused on advanced or unresectable gastric cancer. We considered conventional cutoff values of CA125 to be insufficient to predict the recurrence and prognosis of gastric cancer. Therefore, in our study, we established new cutoff values, which demonstrated a significant correlation with recurrence and prognosis of patients with gastric cancer. Using the new cutoff value, a preoperative CA125-positive status demonstrated a significant correlation with TNM stages, which increased the unresectable rate. This partially indicates CA125 as an independent poor prognostic factor. Huang et al. also showed results with cutoff setting close to ours [18].

**Table 4:** Summary of previous studies related to CA125 in patients with gastric cancer Yes, significant; NA, not applicable CA125, carbohydrate antigen 125; T, depth of tumor invasion; N, lymph node metastasis; M, distant metastasis; P, peritoneal metastasis

Author	Year	Number of patients	Preoperative CA125-positive rate (%)	CA125 cutoff (IU/mL)	Mentioned the relationship of cancer progression				Prognostic impact	Postoperative monitoring
					T	N	M	P		
Hu et al. [11]	2022	472	9.3	35	NA	NA	NA	NA	Yes	NA
Li et al. [17]	2022	268	NA	20.4	NA	NA	Yes	NA	NA	NA
Bao et al. [7]	2022	1264	0.7-28.2	12.2	NA	NA	NA	Yes	Yes	NA
Huang et al. [18]	2019	344	8.3-41.6	17.3	NA	NA	NA	Yes	NA	NA
Namikawa et al. [8]	2018	245	34.3	35	NA	NA	NA	Yes	Yes	NA
Wang et al. [6]	2016	1692	42	13.7	Yes	Yes	Yes	NA	Yes	NA
Kim et al. [10]	2015	679	5.1	37	NA	NA	Yes	Yes	Yes	NA
Present study	2024	525	25	Preoperative: 15.7	Yes	Yes	Yes	Yes	Yes	Yes
				Postoperative: 17.3						

The malignancy of gastric cancers can now be more accurately predicted with the new cutoff value. The notably higher CA125 positive rate in advanced gastric cancer is likely attributed to the frequent occurrence of peritoneal dissemination and the influence of latent peritoneal dissemination [17]. CA125 is derived from endothelial cells in the peritoneum and is believed to be strongly associated with peritoneal dissemination in several other cancers, including gastric cancer [19,20]. Furthermore, the difficulty in the preoperative diagnosis of peritoneal dissemination is believed to be significantly related to the noncurative resection rate [10]. In this study, CA125-negative group demonstrated a better prognosis than CA125-positive group, even with peritoneal dissemination. The prognostic curves of CY1/CA125(−) and CY0/CA125(+) were similar, although the prognosis of CY1 was generally poor, revealing that CA125 (+) may be a prognostic factor similar to CY1. This indicates occult dissemination that cannot be determined by CY or dissemination is likely to progress because of persistent peritoneal inflammation. This is because an increase in CA125 indicates peritoneal damage and inflammation, as well as the establishment of cancer cell dissemination through transplantation [19,20].

Furthermore, many studies focused on preoperative CA125 and prognosis for gastric cancer, but none of the studies reported the correlation between postoperative changes and prognosis. To the best of our knowledge, our study is the first to evaluate a new CA125 cutoff value to assess the association between recurrence and prognosis. CEA or CA19-9 levels usually decreased postoperatively, but CA125 levels frequently increased postoperatively. This is largely dependent on the temporary release of CA125 due to surgically related peritoneal destruction in contrast to other tumor markers. We compared the postoperative changing patterns of CA125 levels between the recurrence and non-recurrence groups. As shown in Figure 5, our follow-up period of postoperative tumor marker was every three months, and we found that the two curves of the recurrence and non-recurrence group started to separate at three months postoperatively. However, at that time, only univariate analysis showed a significant difference between the two groups, but using this new cutoff value may be useful for predicting early recurrence after three months postoperatively.

To analyze the reason why CA125-positive group of stage I has a slightly poorer prognosis, we investigated the causes of death in stage I patients (data not shown). A total of 67% of causes of death in stage I patients were non-cancer diseases. The mortality rates were 19% in the CA125-positive group and 9% in the CA125-negative group. Because all who died in stage I were male, gender was a poor prognostic factor (Table 2). Cardiovascular disease may be reflected as the cause of death in males. Recent studies on cardiovascular disease revealed an association between CA125 and right heart congestion [21,22]. These results indicate the need to investigate the relationship between CA125 and cardiovascular events in gastric cancer patients.

Our study had several limitations. First, this was a single-center retrospective analysis. The difference between preoperative and postoperative CA125 cutoff values is small. Therefore, further studies are needed to determine whether these values predict recurrence or poor prognosis. Second, the clinical diagnosis of peritoneal dissemination is difficult. However, all cases analyzed in this study underwent surgery, and confirmation was conducted macroscopically and using cytology techniques. Third, the relationship between CA125 and chemotherapy after recurrence has not been investigated. Finally, we recognize the need to further investigate the relationship between CA125 and cardiovascular events in patients with gastric cancer, including brain natriuretic peptide, which is a heart failure marker, and ejection fraction, which is a heart function indicator.

## Conclusions

High preoperative CA125 (>15.7 IU/mL) was a significant predictor of noncurative resection and poor overall survival in gastric cancer. Postoperative CA125-positive status (>17.3 IU/mL) at three months postoperatively may also be a potential predictor of recurrence and poor prognosis. Therefore, neoadjuvant chemotherapy should be considered for gastric cancers that exceed this new preoperative CA125 cutoff value.
